# The Novel Impact of Augmented Reality and 3D Printing in the Diagnosis of Complex Acetabular Fractures: A Comparative Randomized Study in Orthopedic Residents

**DOI:** 10.3390/jcm13113059

**Published:** 2024-05-23

**Authors:** Marco Montemagno, Gianluca Testa, Flora Maria Chiara Panvini, Gianluca Puglisi, Giacomo Papotto, Emanuele Marchese, Vito Pavone

**Affiliations:** 1 Department of General Surgery and Medical Surgical Specialties, Section of Orthopaedics and Traumatology, University Hospital Policlinico G.Rodolico-San Marco, University of Catania, 95123 Catania, Italy; docmontemagno@gmail.com (M.M.); flo.panvini@gmail.com (F.M.C.P.); gianlucapuglisi1992@hotmail.it (G.P.); manumarchese1@hotmail.it (E.M.); vitopavone@hotmail.com (V.P.); 2 Department of Orthopaedics and Traumatology, Emergency Hospital Cannizzaro, 95123 Catania, Italy; giacomopapotto@gmail.com

**Keywords:** augmented reality, 3D printing, acetabular fracture, diagnostic accuracy, orthopedic residents

## Abstract

Augmented reality (AR) and 3D printing (3DP) are novel technologies in the orthopedic field. Over the past decade, enthusiasm for these new digital applications has driven new perspectives in improving diagnostic accuracy and sensitivity in the field of traumatology. Currently, however, it is still difficult to quantify their value and impact in the medical–scientific field, especially in the improvement of diagnostics in complex fractures. Acetabular fractures have always been a challenge in orthopedics, due to their volumetric complexity and low diagnostic reliability. **Background/Objectives**: The goal of this study was to determine whether these methods could improve the learning aspect and diagnostic accuracy of complex acetabular fractures compared to gold-standard CT (computed tomography). **Methods**: Orthopedic residents of our department were selected and divided into Junior (JUN) and Senior (SEN) groups. Associated fractures of acetabulum were included in the study, and details of these were provided as CT scans, 3DP models, and AR models displayed on a tablet screen. In a double-blind questionnaire, each resident classified every fracture. Diagnostic accuracy (DA), response time (RT), agreement (R), and confidence (C) were measured. **Results**: Twenty residents (JUN = 10, SEN = 10) classified five fractures. Overall DA was 26% (CT), 18% (3DP), and 29% (AR). AR-DA was superior to 3DP-DA (*p* = 0.048). DA means (JUN vs. SEN, respectively): CT-DA was 20% vs. 32% (*p* < 0.05), 3DP-DA was 12% vs. 24% (*p* = 0.08), and AR-DA was 28% vs. 30% (*p* = 0.80). Overall RT was 61.2 s (±24.6) for CT, 35.8 s (±20.1) for 3DP, and 46.7 s (±20.8) for AR. R was fairly poor between methods and groups. Overall, 3DPs had superior C (65%). **Conclusions**: AR had the same overall DA as CT, independent of experience, 3DP had minor differences in DA and R, but it was the fastest method and the one in which there was the most confidence. Intra- and inter-observer R between methods remained very poor in residents.

## 1. Introduction

Acetabular fractures are complex and require accurate preoperative identification to determine an optimal treatment plan due to the difficult surgical approach and the need to determine the most suitable fixation technique. Acetabular fractures are identified with the aid of the worldwide-recognized Judet and Letournel classification system [1,2].

Although it seems old, this classification remains the most used in the identification of these fractures and CT scans nowadays represent the gold standard for emergency room radiologic wards to assess the proper planning for these fractures [3].

Before the advent of the CT scan, standard X-rays allowed us to visualize and understand the entity of lesions, fragmentations, and dislocations only during open surgery. Understanding the dislocation of fragments, the amount of displacement, and the joint involvement is necessary for successful treatment. CT has proven to be superior to conventional X-rays in the evaluation of articular fractures, affording optimal anatomical assessment in the axial, coronal, and sagittal planes [4].

These two-dimensional (2D) imaging modalities are used to interpret acetabular fractures. However, the complex three-dimensional (3D) geometry and relatively low incidence of these fractures can make it difficult for residents to become adept at correctly identifying acetabular fractures [5].

New horizons have been explored with the birth of 3D technology. 3DP models for orthopedic conditions can generate accurate reproductions of anatomical structures, with faithful representations of both normal and pathologic variations. Some current surgical applications of 3DP [6] include preoperative templating, deformity correction planning, custom prosthesis printing, discussions with patients, and trainee education [7,8,9].

It has been demonstrated that the use of 3D CT imaging has significantly improved resident accuracy in acetabular-fracture identification. Also, an improvement from fair to moderate inter-observer agreement when residents used 2D versus 3D CT images to identify acetabular fractures has been observed [10]. On the other hand, the current COVID-19 pandemic has improved the use of AR technology [11].

Different from virtual reality, AR technology provides a computer-generated overlay onto real-world surfaces, providing depth perception and stereoscopic visualization to the user [12,13].

AR has also benefited from recent advancements in handheld smartphone and tablet devices, which not only improve the power of such programs but also expand their accessibility out of traditional learning spaces and into the hands of the learner [14,15]. AR is a rapidly developing technology. Due to its flexibility in integrating physical and virtual environments, AR-based programs are increasingly used in education, including medical education and training. The use of this technology provides various means of delivering learning content and enhancing students’ experiences, diagnosis, and surgical planning.

The purpose of this study was to compare the diagnostic accuracy between the new 3D technologies (AR and 3DP) to the gold-standard CT scans in the classification of complex acetabular fractures among orthopedic residents. The second purpose was to investigate the confidence levels and the differences based on years of experience.

Approval was obtained from the ethics committee of our university. The procedures used in this study adhered to the tenets of the Declaration of Helsinki.

## 2. Materials and Methods

### 2.1. Patients

Twenty orthopedic residents of our department were selected and divided by the year of the course. 

First-year and second-year post-graduate residents were grouped in the Junior (JUN) group, and third-, fourth-, and fifth-year post-graduate residents were grouped in the Senior (SEN) group. Fractures of the acetabulum of patients admitted to our unit from 2015 to 2021 were included in the study; the inclusion criteria were as follows: associated fracture of acetabulum according to the Letournel classification and no associated or pathological fracture (osteoporotic bone, tumor, or metabolic syndrome). Informed consent was obtained from all individual participants included in the study.

### 2.2. Methods

**Figure 1:**
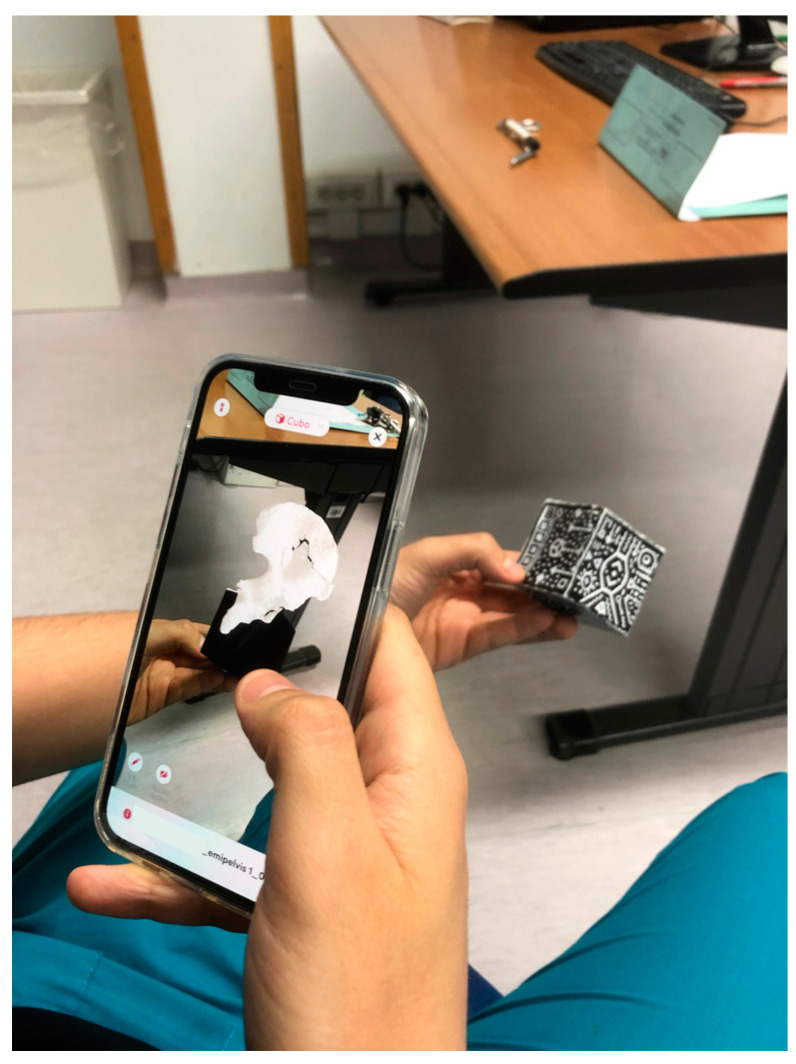
AR model in screen visualization with Object Viewer app.

For each acetabular fracture used in the study, we provided:

- CT scan (1mm layer thickness, 1 mm layer interval, and a voltage of 120 kV);
- 3DP model obtained from DICOM file using 3D planning software (Mimics-Materialise v.24.0, Meshmixer v.3.5, Cura v.5.6) with the use of segmentation applying the “region growing” function to separate the bones and soft tissues, define the fracture fragments, and build a hemipelvis digital model for each fracture that was printed in polylactic acid (PLA) (scale 1:2) using a high-precision 3D printer (Ultimaker © 2+ extended, Ultimaker, Netherland);
- 3D digital rendered model (format .stl) displayed in AR with a tablet screen using Object Viewer app (by Merge EDU ©, Merge Labs Inc., San Antonio, TX, USA) Figure 1.

### 2.3. Methods of Assessment

Associated acetabular fractures were properly classified by three senior surgeons specialized in pelvic trauma, and with more than ten years of experience in the use of the Letournel classification. The final assessment of the type of fracture was carried out . in open surgery during treatment. Simple acetabular fractures were excluded from this study.

In a double-blind study, each resident was interviewed with a questionnaire to classify every fracture using each proposed method. A web-based questionnaire for each resident was completed in which we collected:

- The numeric fracture classification according to the Letournel classification (1: anterior wall, 2: anterior column, 3: transverse, 4: posterior wall, 5: posterior column, 6: posterior wall + posterior column, 7: anterior column + posterior hemitransverse, 8: T-shaped, 9: transverse + posterior wall, and 10: both columns);
- The response time for the correct answers;
- Answers to six Likert-scale survey questions about perceived confidence in each diagnostic method.

From these data, we calculated the diagnostic accuracy (DA, as a percentage of correct answers), the response time calculated only for the correct answers (RT), and the confidence (C) sorted by: groups, overall, and diagnostic method. 

Before starting the questionnaire, each resident was briefly presented with a diagram of acetabular fractures sorted by number, and then a short course on how to read a 3D object from the augmented reality application was provided.

No residents enrolled in the test had previous familiarity with 3D-printing or augmented-reality technology.

### 2.4. Statistical Analysis

Data were processed statistically using mean, standard deviation, minimum, maximum, Fisher’s exact test, t-student (significance *p* < 0.05), and Fleiss’ Kappa guidelines for inter- and intra-observer reliability (R) between methods and groups. All data were processed and graphs (box plots and histograms) were made using Microsoft Excel (type 16.85) and SPSS (IBM, 29.0) software.

## 3. Results

A total of 20 trainees from of the residency program of our school were selected and enrolled in the study (five from PGY-1, two from PGY-2, three from PGY-3, four from PGY-4, and three from PGY-5). 

**Figure 2:**
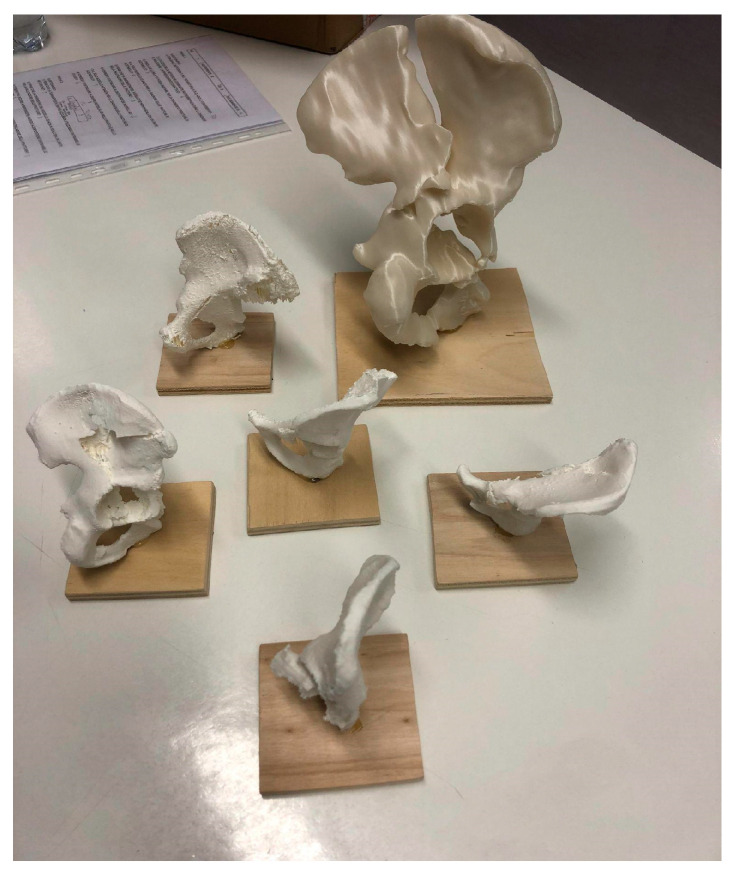
3DP PLA (scale 1:2) fracture models used in the study.

The mean age was 28.6 years (±2.3), with an M:F ratio of 17:3. They were divided into JUN (*n* = 10) and SEN (*n* = 10) groups. Five associated fractures of the acetabulum (according to the Letournel classification) were included, and, for each fracture, a CT scan, 3D printed (3DP) model (Figure 2), and augmented reality (AR) model were provided. Results were collected using the questionnaire described above.

Overall, DA means between diagnostic methods were 26% (±0.14) for CT, 18% (±0.15) for 3DP, 29% (±0.30) for AR, and 23% (±0.20).

CT-DA was higher than 3DP-PA (*p* = 0.05), AR-DA was superior to 3DP-DA (*p* = 0.048), and no statistically significant difference was found between CT-DA and AR-DA (*p* = 0.64) (Table 1).

As shown in Table 2, DA between JUN and SEN was as follows: CT, JUN-DA 20% (±0.09) and SEN-DA 32% (±0.17) (*p* = 0.05); 3DP, JUN-DA 12% (±0.17) and SEN-DA 24% (±0.13) (*p* = 0.08); AR, JUN-DA 28% (±0.21) and SEN-DA 30% (±0.23) (*p* = 0.845); overall, JUN-DA was 17.8% (±0.09) and SEN-DA 29% (±0.13) (*p* = 0.033).

Overall RT to obtain the correct answer was 61.2 s (±24.6) for CT, 35.8 s (±20.1) for 3DP, and 46.7 s (±20.8) for AR.

RT for the JUN and SEN groups was as follows: CT, JUN-RT was 48.1 s (± 24.5) and SEN-RT 69.5 s (± 21.6) (*p* = 0.07); 3DP, JUN-RT was 39 s (±0.17) and SEN-RT 34.3 s (+21.9) (*p* = 0.717); AR, JUN-RT was 46.5 s (±23.4) and SEN-RT 46.9 (±19.2) (*p* = 0.958).

Overall intra- and inter-observer R was fairly poor between methods and the groups of residents (JUN k = 0.06, SEN k = 0.09).

**Figure 3:**
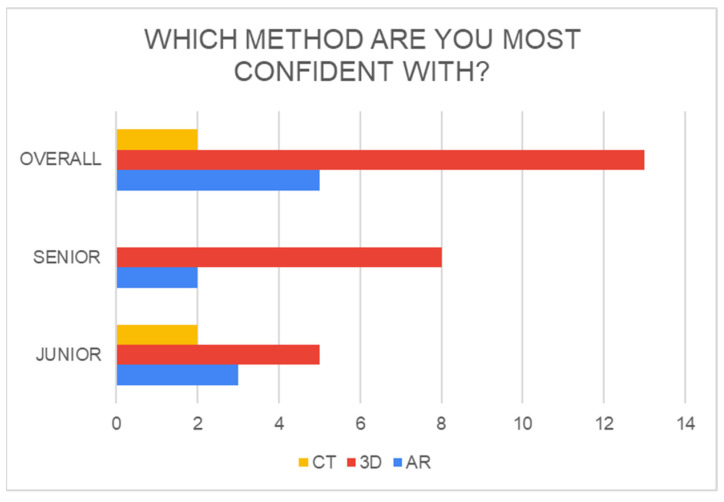
Confidence in diagnostic methods between groups in the method evaluation questionnaire.

**Figure 4:**
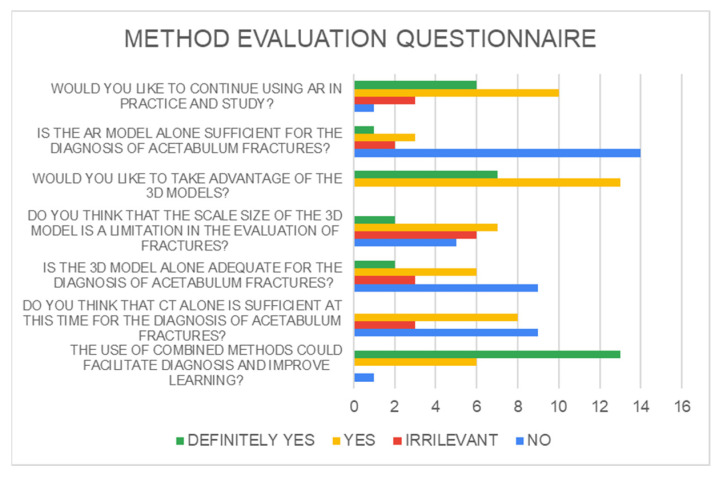
Method evaluation questionnaire.

3DP models provided superior results overall C (65%), even if answering for only AR and 3DP alone was not enough to make the diagnosis (results are shown in histograms, Figure 3 and Figure 4).

## 4. Discussion

CT imaging has proven to be superior to conventional X-rays in the evaluation of complex articular fractures, allowing one to understand the entity of lesions, the fragmentations, and the dislocations, affording optimal anatomical assessment in the axial, coronal, and sagittal planes [16]. 3D volume rendering from CT has led to better accuracy and rendered acetabular fracture diagnosis more repeatable and faster [17]. The need to improve the “Letournel accuracy” in residents with new diagnostic methods is an important topic in the literature [18], using a different method for teaching (3D models, algorithms) [19,20,21,22,23].

The advent of new technologies, 3DP and AR, or e-learning as well, in recent years, has increasingly gained importance in student learning and practice [4,24]. The incorporation of 3D models has been shown to be useful for resident education in surgical specialties such as neurological, thoracic, plastic, and general surgery [5,25]. Three-dimensional volume rendering reconstruction helps visualize the sense of spatial anatomy and, likewise, pathology. This visionary process is described by many authors of different surgical and clinical specialties, not just for traumatology planning purposes, [8,9] but also for purposes from vascular neurosurgery to lung imaging [26,27,28].

In recent years, several orthopedics papers [7,29,30,31] have demonstrated the effectiveness of using 3D printed models in the trauma field of large joint fractures such as tibial-plateau and acetabular fractures, where the use of pre-formed plates and planned cutting masks used in the operating room makes a difference to outcomes.

Conversely to Lim et al. [5], and as well as Montgomery et al. [32] we properly chose five acetabular complex fracture patterns, thinking that 2D CT would be adequate in the classification of the other types.

Lim et al. [5] in their study demonstrated an improved resident accuracy in acetabular fracture identification with the use of 3D models compared with X-rays. When comparing 3D models to CTs, the participants obtained similar accuracy with each modality, and both were significantly superior to X-rays. However, there was no significant difference between CT and 3D models.

In our study, we compared the use of CT, 3DP models, and AR models. To our knowledge, no study has previously evaluated the importance of AR technologies compared to 3D or CT technologies in the diagnostic evaluation and classification of fracture patterns.

Contrary to Lim et al. [5], when comparing 3D models to CTs, the participants obtained different accuracy with each modality. The use of 3DP models led to lower accuracy in the classification of acetabular fractures, maybe due to the fact that staff could downgrade the severity of fracture classification. We can maybe blame this problem on the difficult reproduction or interpretation of the details of the more complex fracture rhymes, as the 3DP model cannot reproduce these every time [33].

Despite this result, senior and junior residents agreed that they felt more confident and comfortable with 3D models. The addition of a 3DP model had no proven benefit for residents in their final year of training.

This fact can be associated with a better approach and 3D-tactile proprioception with the object and, therefore, the tridimensional complexity of the fracture.

The overall accuracy of the surgeons-in-training remained different between the groups, especially with this type of fracture. Senior residents with more experience in orthopedics had a higher overall DA.

**Figure 5:**
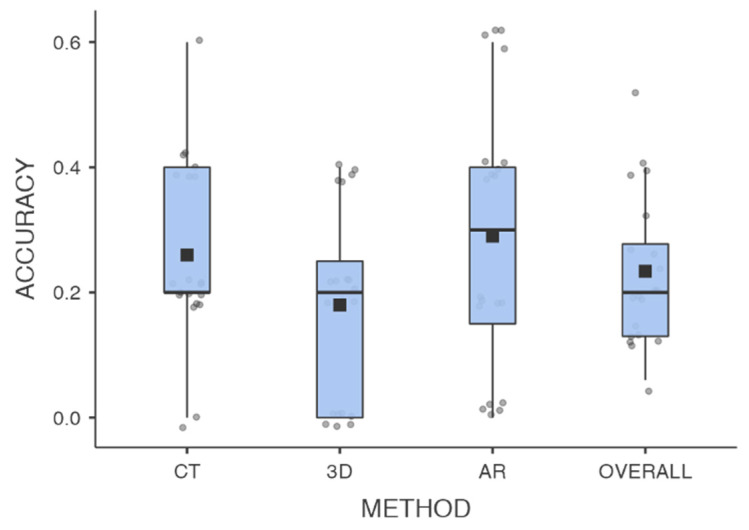
Box plots showing DAs between methods in residents’ evaluations of acetabular fractures in this study (CT: CT scan; 3D: 3D printed models; AR: augmented-reality digital models).

Our study showed that AR seemed to be successful as a CT scan method in diagnostic accuracy (Figure 5).

Junior residents had better accuracy when using AR and this method succeeded in eliminating the JUN–SEN groups’ differences in accuracy in the recognition of these fractures. This was the most effective and best statistically demonstrated result of this study. There are two reasons for this result: the first is related to the interactive immediacy of visualization in AR—it stands out compared with the simple volumetric display on 2D screen because it integrates the display in a “real space” that is represented by the interaction of the object with the camera (literally an “augmented” reality). This makes the fracture more explorable and the method is more intuitive even for less-experienced surgeons. The second reason is probably linked to the new generations of surgeons; they are much more dependent on screens and digital visualizations, which makes the approach to technology more immediate and effective.

However, the fastest method, albeit less accurate, seemed to be 3DP.

Differently from the literature [34,35,36], Letournel’s classification still had a poor interobserver agreement (k < 0.100), and our study did not find an improvement in reliability using these new technologies.

A study by Matta et al. [35] assessed good reliability for this classification only for senior surgeons who have been taught how to interpret the images and who regularly treated acetabular fractures.

Subjectively, 65% of the residents agreed or strongly agreed that they felt confident identifying acetabular fractures before interacting with the 3DP models. However, over 95% agreed or strongly agreed that exposure to the 3D models combined with the AR improved their confidence in fracture identification. This result is due to their lack of experience or exposure to the new method.

Participants also indicated that the visual appearance of the models was an accurate representation of the fractures, but the size was the real limitation. Furthermore, 80% of respondents stated that they either agreed or strongly agreed that they would like to continue using 3D models in other areas of their education. Overall, the subjective data support the notion that 3D models are perceived as a positive addition to orthopedic resident training. AR seemed to be the most intuitive method. Some works argue for the use of both AR and VR technologies in orthopedic trauma surgery, highlighting, specifically, the combination of AR with 3DP. A direct empirical comparison of these technologies has been carried out [31]. More studies should be carried out, making augmented reality, combined with 3DP technology, an increasingly used tool in learning, diagnostics, and pre-operative planning.

In considering the results of this study, the future prospects of these AR technologies will surely include the implementation of AR tools in the operating room, leading to a technology transition to a mixed reality in elective surgery (arthroplasty, osteotomies, and corrections deformity) and orthopedic traumatology.

### Limitations of the Study

The cohort of fractures included in this study will be improved in future research, although the results obtained between methods were statistically valid.

The 1:2 scale model may have affected the correct visualization for the observers compared with a perception of a life-size model but the declared detail of the fracture rhymes remained the same for the accuracy of the printer used for the study models.

Certainly, more investigations and studies need to be carried out on a larger scale to introduce augmented reality technology to difficult everyday joint trauma, considering the demonstrated benefit of faster and more accurate identification of the most complex fracture patterns.

## 5. Conclusions

This study demonstrated that AR diagnostic accuracy in acetabular fractures was similar to that of CT models and superior to that of 3DP models. The 3DP models had poor accuracy in the diagnostic process. On the contrary, AR represents a novel technology that evens out differences related to the experience of surgeons. AR must not be the alternative solution to the CT gold standard for the diagnosis of complex joint fractures but can be a better tool for learning and managing fractures in the future of orthopedics.

## Figures and Tables

**Table 1:** General DA differences between methods.

Accuracy Difference	Mean Difference	*t*-Test	*p*
CT vs. AR	−0.30	−0.508	0.61
CT vs. 3DP	0.80	1.66	0.052
3DP vs. AR	0.120	2.04	0.048

CT: CT scan; 3DP: 3D printed models; AR: augmented-reality digital models.

**Table 2:** DA differences between JUN and SEN groups.

	Group	Mean	SD	*t*-Test	*p*
CT Accuracy	JUN	0.200	0.0943	−1.964	0.050
	SEN	0.320	0.169		
3DP accuracy	JUN	0.120	0.1687	−1.800	0.08
	SEN	0.240	0.126		
AR accuracy	JUN	0.280	0.2150	−0.198	0.84
	SEN	0.300	0.236		
Overall accuracy	JUN	0.178	0.0909	−2.267	0.036
	SEN	0.290	0.127		

CT: CT scan; AR: augmented-reality digital models; JUN: Junior group; SEN: Senior group; SD: standard deviation.

## Data Availability

Data are available in the tables of the manuscript.
